# Determination of Curcuminoids, Piperine, Boswellic Acids and Andrographolides in Food and Dietary Supplements by HPLC

**DOI:** 10.17113/ftb.60.04.22.7560

**Published:** 2022-12

**Authors:** Edvin Brusač, Mario-Livio Jeličić, Biljana Nigović, Daniela Amidžić Klarić, Ana Mornar

**Affiliations:** Department of Pharmaceutical Analysis, Faculty of Pharmacy and Biochemistry, University of Zagreb, Ante Kovačića 1, 10000 Zagreb, Croatia

**Keywords:** functional food, quality control, Box-Behnken design, curcuminoids, boswellic acids, andrographolides

## Abstract

**Research background:**

As use of functional food and herbal combination products is ever increasing, methods for quality control of such preparations are necessary. Moreover, low quality of products can cause either lack of benefit or harm to the consumer. In this work, determination of three curcuminoids, piperine, six boswellic acids and three andrographolides, often used in combination products, was carried out in raw materials and dietary supplements.

**Experimental approach:**

After extraction optimization using Box-Behnken design, maximum active substance yields were obtained using 81.5% ethanol in hydroethanolic extraction solvent, 30 min sonication time and 60 °C extraction temperature. Afterwards, a high-performance liquid chromatography method was developed and validated, with special attention paid to selectivity, precision and robustness of the method. Lastly, 54 food and dietary supplement samples were analyzed.

**Results and conclusions:**

Most products were bought locally, from credible vendors and they all complied with relevant regulatory requirements. However, products obtained on the Internet contained little to no active substances (24% of samples contained less than 20% declared content), presumably showing no efficacy, or were either found to be likely adulterated or contained very high amounts of active substances, compromising safety in terms of dose-dependent adverse effects (one sample containing andrographolides) and pharmacokinetic interactions (one sample containing piperine). In conclusion, consumers should refrain from purchasing such products from the Internet and obtain them only from verified suppliers such as local pharmacies or health stores.

**Novelty and scientific contribution:**

This work demonstrates the first developed method for the analysis of aforementioned combination products, which are on the rise today. The method is simple and robust and can be adapted by most laboratories for routine quality control of the said products. Moreover, the work sheds light on the low quality of several products and signifies the need for increased consumer awareness of dangers of taking such products.

## INTRODUCTION

**Fig. S1:**
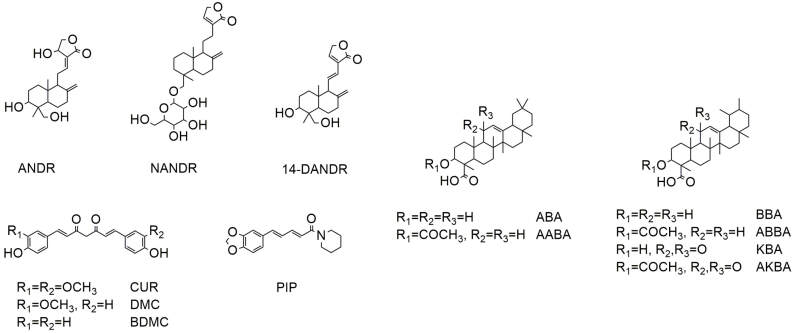
Structures of selected analytes. ANDR=andrographolide, NANDR=neoandrographolide, 14-DANDR=14-deoxy-11,12-didehydroandrographolide, CUR=curcumin, DMC=demethoxycurcumin, BDMC=bisdemethoxycurcumin, PIP=piperine, ABA=α-boswellic acid, AABA=3-*O*-acetyl-α-boswellic acid, BBA=β-boswellic acid, ABBA=3-*O*-acetyl-β-boswellic acid, KBA=11-keto-β-boswellic acid, AKBA=3-O-acetyl-11-keto-β-boswellic acid

Plants and their preparations have been used to treat and prevent a myriad of diseases since time immemorial. Today it is well known that plants contain various substances which exert a pharmacological effect, many of which are the main compounds in drug development. However, with the rise of modern pharmaceutical industry, one would expect a decline in the use of complementary and alternative medicine, but this is not the case; some studies suggest prevalence of herbal medicine use up to 48% in the European Union (*1*-*3*). The use of functional food and herbal dietary supplements in chronic inflammatory conditions such as arthritis, inflammatory bowel disease and asthma is also fairly common as patients consider them to be safe and effective because of their natural origin. Turmeric, Indian frankincense and green chiretta stand out among others because of their widespread use. Rhizomes of turmeric (*Curcuma longa* L., *Zingiberaceae*) contain curcuminoids curcumin (CUR), demethoxycurcumin (DMC) and bisdemethoxycurcumin (BDMC) (Fig. S1), which have been shown to modulate activities of glutathione peroxidase, superoxide dismutase and catalase, as well as block nuclear factor kappa-light-chain-enhancer of activated B cells (NF-κB) activation, thereby displaying anti-inflammatory and antioxidative properties (*4*). Piperine (PIP), an active substance of black pepper (*Piper nigrum* L., *Piperaceae*) fruit enhances the bioavailability of curcuminoids (*5*), so it is often used in combination with turmeric. Indian frankincense (*Boswellia serrata* Roxb. ex Colebr*., Burseraceae*) contains boswellic acids, the most prevalent of which are α- and β-boswellic acids (ABA and BBA), followed by 3-*O*-acetyl-α- and β-boswellic acids (AABA and ABBA), 11-keto-β-boswellic acid (KBA) and 3-*O*-acetyl-11-keto-β-boswellic acid (AKBA). It is thought that boswellic acids are responsible for immunomodulatory and anti-inflammatory properties of frankincense resins and extracts through 5-lipoxygenase, leukocyte elastase, mitogen-activated protein kinase (MAPK) and NF-κB pathway activity modulation, among others (*6*). KBA and AKBA are thought to be most pharmacologically potent in this regard (*7*). Diterpene lactones andrographolide (ANDR), neoandrographolide (NANDR) and 14-deoxy-11,12-didehydroandrographolide (14-DANDR), active constituents of green chiretta (*Andrographis paniculata* (Burm. f.) Wall. ex Nees, *Acanthaceae*) are presumed to exert anticancer, anti-inflammatory, immunomodulatory and other effects through interleukine reduction, matrix metalloproteinase and growth factor suppression, NF-κB and Janus tyrosine kinase activity modulation, *etc*. (*8*).

In terms of all products, herbal supplements being no exception, the efficacy is questioned if the content of active substances is lower than expected. Conversely, higher than expected amounts of active substances could lead to overdose and toxicity, as well as an increased risk of pharmacokinetic or pharmacodynamic interaction with concomitantly used conventional therapy (*9*). It follows that the bioactive compound content should be well established and accurate, complying with the declaration. Additionally, active substance acceptance limits for processed botanical forms and herbal preparations are established in monographs of many pharmacopoeias, United States Pharmacopeia (USP) and European Pharmacopoeia (Ph. Eur.) being some of the most relevant (*10*, *11*). Several studies have revealed major discrepancies between the labelled and found content of herbal dietary supplements (*12*-*15*), which is even more emphasized in the products bought from dubious sources. Products purchased *via* the Internet are rarely subjected to quality assessment by regulatory agencies and are adulterated with active pharmaceutical ingredients or contain inaccurately labelled amounts of active substances (*16*, *17*). This, in turn, signifies stricter control of active substance content of such products is necessary.

Since some components can demonstrate pharmacological synergism by, for example, enhancing bioavailability or potentiating the pharmacodynamic effect of other mixture components, it is of no surprise that herbal mixture formulations are becoming more popular in phytomedicine (*18*), just as fixed-dose combinations are in standard pharmacotherapy. Although combination products of the aforementioned herbal drugs and their preparations are also becoming more prevalent for the same reasons, according to our findings no analytical method for simultaneous determination of all above-mentioned active substances has been developed yet. Methods for quantification of curcumin and piperine have been developed (*19*, *20*), albeit not mentioning or determining BDMC and DMC. HPLC and HPTLC methods for determination of curcumin and α- and β-boswellic acids (*21*) and curcumin, PIP and boswellic acid (*22*) have been developed, but the separation of the components of each substance group has not been achieved, while there is no literature data about methods for the determination of BDMC and DMC, or other boswellic acids. Therefore, this work aims to firstly develop and validate an HPLC method for simultaneous determination of three andrographolides, three curcuminoids, six boswellic acids and PIP as most potent active compounds and subsequently utilize it for quality control of mono- or combination products of the respective botanicals available locally and from the Internet.

## MATERIALS AND METHODS

### Chemicals and reagents

Andrographolide (≥ 98.0%) was obtained from TCI (Tokyo, Japan), while all the other active substance standards (standard analytical grade) were purchased from Sigma-Aldrich, Merck (St. Louis, MO, USA). Formic acid (LiChropur, 97.5–98.5%) and ethanol (LiChrosolv, gradient grade) were supplied by Supelco (Bellefonte, PA, USA). Acetonitrile (HPLC grade) was purchased from Avantor (Radnor, PA, USA). Ultrapure water was produced using an Ultra Clear UV water purifying system (SG Water, Barsbuttel, Germany), resistivity >18 MΩ/cm at 25 °C and total organic carbon <5 µg/mL. Excipients for the selectivity testing were hydroxypropyl methylcellulose Methocel K100M Premium CR (Colorcon, Harleysville, PA, USA), stearic acid, lactose monohydrate, wheat, rice and corn starch (Kemig, Zagreb, Croatia) and magnesium stearate (Acros Organics, Princeton, NJ, USA).

### Samples

In total, 54 samples (raw material, food and dietary supplements), of which 35 preparations contained extracts, while 19 contained processed botanical forms and botanical products. From local pharmacies 13 samples were procured, 15 from food health stores and 26 were purchased from the Internet (products available online only in Croatia). The samples were coded with letter S followed by the corresponding sample number. All samples were analyzed prior to the stated expiry date. A detailed description of all analyzed samples, including mode of acquisition, manufacturer origin, sample type and label, is given in Table S1.

### Instrumentation

Weighing of masses below 100 mg was done on an MX5 Microbalance with a readability of 1 µg, while those above 100 mg were weighed using an AG245 balance, both from Mettler Toledo (Columbus, OH, USA). Extraction procedure was done using an Elmasonic xtra TT ultrasonic bath (Elma Schmidbauer GmbH, Singen, Germany). Centrifugation was conducted on a mini G centrifuge (IKA, Staufen im Bresgau, Germany) and a centrifuge with temperature control Z 326K (Hermle, Gosheim, Germany) at 1200×*g*. Analyses were carried out on an Agilent 1260 series chromatograph equipped with a binary pump, degasser, autosampler, column oven and diode array detector operated using Chemstation OpenLab CDS rev. C01.10 (Agilent Technologies, Santa Clara, CA, USA).

### Chromatographic analysis

Separation was conducted on an HSS Cyano column, 150 mm×3.0 mm, 3.5 µm particle size (Waters Corporation, Milford, MA, USA) thermostated at 40 °C. Ultrapure water and acetonitrile, both acidified with final content of 0.1% formic acid, were used as mobile phase components A and B, respectively. Gradient elution at a flow of 1 mL/min was applied as follows: 0–6 min isocratic 40% B, 6–16.5 min linear gradient 40–70% B, 16.5–17.5 min linear gradient 70–100% B, and 17.5–18 min isocratic 100% B. Pure organic phase was applied for two more minutes and the column was equilibrated to starting conditions with the total method run time of 25 min. Injection volume was set to 5 µL and the needle was washed prior to each injection with methanol . Autoinjector temperature was 15 °C. Detection wavelengths were set to 206 (for ABA, BBA, AABA, ABBA and NANDR), 230 (for ANDR), 256 (for 14-DANDR, KBA and AKBA), 340 (for PIP) and 422 nm (for BDMC, DMC and CUR), bandwidth 4 nm, no reference wavelength.

### Identification of AABA and ABBA using mass spectrometry

AABA and ABBA identification in real samples was conducted using Synapt G2-Si ESI-QTOF-MS system, controlled using MassLynx v. 4.1 software (Waters Corporation). MS conditions were as follows: sampling cone voltage 60 V, source temperature 120 °C, desolvation temperature 350 °C, capillary voltage 3 kV, and desolvation gas flow 600 L/h. Spectra were acquired in positive ion mode. MS/MS experiments were done using *m/z*=499.4 as precursor and collision energy of 5 V, scanning from 100 to 500 *m/z*.

### Optimization of extraction procedure using response surface methodology

Ultrasound-assisted extraction of active substances was optimized using response surface methodology approach, more specifically a three-factorial Box-Behnken design. The methodology was applied to a mixture of processed botanical forms (*m*(powdered green chiretta leaf):*m*(powdered Indian frankincense resin):*m*(powdered turmeric rhizome):*m*(powdered black pepper fruit)=20:10:2:1). A mass of 25 mg of mixture was suspended in 10 mL of solvent and subjected to ultrasonic extraction at designated temperature and duration. Independent variables were ethanol ratio in the hydroethanolic extraction solvent (40–100%), extraction temperature (30–80 °C) and sonication time (10–30 min). Considering the physicochemical similarities within compounds of the same botanical source, the responses were the sums of extraction yields of all analytes for the respective herbal substance.

### Sample preparation

Samples were extracted using optimized conditions predicted *via* Box-Behnken design. Firstly, the contents of six dosage forms (tablets or capsules) were individually weighed and thoroughly homogenized in a ceramic mortar. For samples in bulk, this step was omitted. A mass of 25 mg of sample was accurately weighed and suspended in 25 mL of 81.5% ethanol (*V/V*), sonicated for 30 min at 60 °C and centrifuged at 1200×*g*. The supernatants were diluted, if necessary, and injected into the HPLC system. Liquid samples were injected *post* centrifugation and appropriate dilution.

### Method validation

The method was validated according to International Council on Harmonization (ICH) guidelines (*23*). The examined parameters included selectivity, linearity, limits of detection (LOD) and quantification (LOQ), precision, accuracy, stability and robustness. The used model samples were previously mentioned processed botanical mixture (for composition, *vide supra*) and dry extract mixture (*m*(S31):*m*(S26):*m*(S21):*m*(S4)=20:5:5:1).

### Statistical analysis

Statistical analyses were done using Microsoft Excel v. 16.0.14026.20270 (*24*). Plackett-Burman robustness testing and Box-Behnken extraction optimization were conducted using Design Expert v. 7.0.0 (*25*).

## RESULTS AND DISCUSSION

### Chromatographic method development

**Fig. 1:**
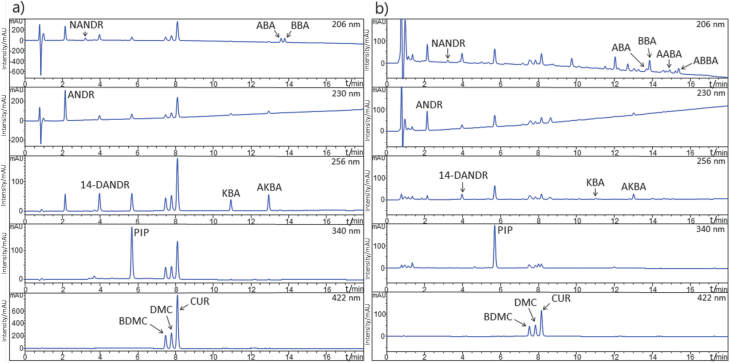
Representative chromatograms of: a) standard solution, and b) botanical processed mixture. ANDR=andrographolide, NANDR=neoandrographolide, 14-DANDR=14-deoxy-11,12-didehydroandrographolide, PIP=piperine, BDMC=bisdemethoxycurcumin, DMC=demethoxycurcumin, CUR=curcumin, KBA=11-keto-β-boswellic acid, AKBA=3-*O*-acetyl-11-keto-β-boswellic acid, ABA=α-boswellic acid, BBA=β-boswellic acid, AABA=3-*O*-acetyl-α-boswellic acid, ABBA=3-*O*-acetyl-β-boswellic acid

Before starting method development, distribution coefficients (log *D*) of analytes were examined to gain insight into their chromatographic behaviour at different pH of mobile phase. Higher log *D* values for all analytes but PIP were observed at low pH, subsequently leading to stronger retention in nonpolar stationary phases. Application of low pH mobile phases would allow for better separation of hydrophilic matrix components from the analytes, enhancing selectivity. Thus, an acidic mobile phase modifier with 0.1% formic acid was chosen. Regarding stationary phases, multiple column packings were tested: C4 (Kromasil, 150 mm×4.6 mm, 3.5 µm particle size, Nouryon, Amsterdam, the Netherlands), C8 (Kinetex, 150 mm×4.6 mm, 2.6 µm particle size, Phenomenex, Torrance, CA, USA), C18 (Hypersil GOLD, 150 mm×4.6 mm, 3 µm particle size, Thermo Fisher Scientific, Waltham, MA, USA), phenyl (CORTECS Phenyl, 150 mm×4.6 mm, 2.7 µm particle size, Waters Corporation) and cyano (HSS Cyano, 150 mm×3.0 mm, 3.5 µm particle size, Waters Corporation). The use of methanol as organic modifier yielded no resolution between the three curcuminoids, which coeluted within a single peak. Additionally, the stated peak severely fronted, up to two minutes prior to its apex. The use of acetonitrile improved the fronting, as well as produced adequate resolution between curcuminoids, although the resolution between CUR and PIP proved unsatisfactory on all columns (lower than 0.89) but HSS Cyano. Moreover, the use of nonpolar columns required 100% organic modifier in the mobile phase to elute the highly lipophilic boswellic acids. Using HSS Cyano column and acetonitrile as a modifier, all peaks were successfully resolved. Boswellic acids also eluted fairly rapidly (under 16 min) and used less organic modifier than in other columns owing to higher polarity of the stationary phase, improving time- and cost-efficacy of the method. After the optimization of flow rate, gradient (mobile phase components A and B were ultrapure water and acetonitrile acidified with 0.1% formic acid, respectively), column temperature and wavelength, the final method was developed with a total run time of 25 min. A representative chromatogram of the standard solution is shown in Fig. 1a. The resolution of the peaks in the standard chromatogram was higher than 1.53 (ABA-BBA pair). In real samples (representative chromatogram of processed botanical mixture is shown in Fig. 1b), resolution between the ABA-BBA pair, as well as among AABA, ABBA and matrix components was observed to be lower than 1.5 (0.90, 1.35 and 1.23 for ABA-BBA, AABA-matrix component and ABBA-matrix component pairs, respectively); however, this was not expected to alter the results markedly.

### Extraction optimization

Active substance extraction using ultrasound was optimized. As the representative sample, a mixture of processed botanical forms as the more complex sample type to extract was chosen. Seeing that all active substances are moderately to highly lipophilic, the choice of organic solvent is crucial. Therefore, methanol, acetonitrile and ethanol in various volume ratios in hydro-organic extraction solvent (20, 60 and 100%, *V/V*) were tested. The lowest yields of all compounds were in methanol. Acetonitrile showed similar yields for curcuminoids, PIP and andrographolides to ethanol, but was deemed inferior in the extraction of boswellic acids. Therefore, ethanol was chosen as the organic modifier in the extraction solvent. Afterwards, a three-factorial Box-Behnken design was employed to maximize the active substance extraction yield from the samples. As the independent variables (factors), volume ratio of ethanol in the hydroethanolic extraction solvent (40–100%), extraction temperature (30–80 °C) and sonication time (10–30 min) were selected. The dependent variables (responses) were extraction yields of active substances (in mg/g of processed botanical mixture). Table 1 shows the experimental design, as well as factors and responses.

**Table 1:** Box-Behnken design with factors and measured responses

Standard	X_1_ *φ*(EtOH)/%	X_2_ Extraction temperature/°C	X_3_ *t*(sonication)/min	*w*(andrographolide)/(mg/g)^a^	*w(*piperine)/(mg/g)^a^	*w*(curcuminoid)/(mg/g)^a^	*w*(boswellic acid)/(mg/g)^a^
16	70	55	20	12.43	1.32	3.05	71.41
2	100	30	20	8.61	1.28	2.32	71.13
7	40	55	30	12.58	1.25	2.53	23.39
9	70	30	10	10.22	1.22	2.41	47.37
8	100	55	30	11.33	1.39	2.85	75.96
11	70	30	30	12.09	1.47	3.03	73.01
12	70	80	30	12.45	1.47	3.21	74.46
10	70	80	10	11.79	1.54	3.02	72.02
6	100	55	10	9.88	1.39	2.55	81.35
4	100	80	20	11.90	1.29	2.81	71.39
1	40	30	20	12.12	1.13	2.00	13.70
14	70	55	20	12.65	1.64	2.91	69.56
15	70	55	20	12.58	1.28	3.11	73.29
17	70	55	20	11.71	1.49	2.80	62.94
3	40	80	20	11.78	1.18	2.27	18.58
13	70	55	20	12.60	1.37	3.11	69.45
5	40	55	10	12.45	1.08	1.85	6.57

^a^Expressed in mg of examined analytes per g of processed botanical mixture

Upon analyzing the data, it was deduced that the reduced quadratic models best describe the extraction yields. The model equations are as follows:

*Y*(andrographolide)=11.527–0.011X_1_**+0.048X_2_**–0.026X_3_**+1.210·10^-3^ X_1_X_2_**+1.097·10^-3^ X_1_X_3_–7.673 ·10^-4^ X_1_^2^**–9.829·10^-4^ X_2_^2^** /1/

*Y*(piperine)=–0.276+0.030X_1_**+0.008X_2_+0.022X_3_–3.194·10^-4^ X_2_X_3_–1.934 ·10^-4^ X_1_^2^** /2/

*Y*(curcuminoid)=–2.789+0.101X_1_**+0.032X_2_**+0.068X_3_**–3.109·10^-3^ X_1_X_3_*–4.349·10^-4^ X_2_X_3_–6.182·10^-4^ X_1_^2^**–1.385·10^-4^ X_2_^2^ /3/

*Y*(boswellic acid)=–195.758+4.924X_1_**+0.620X_2_**+3.066X_3_**–0.019X_1_X_3_**–0.023X_2_X_3_**–0.025X_1_^2^** /4/

where X_1_ is the volume ratio of ethanol in the hydroethanolic extraction solvent, X_2_ is the extraction temperature and X_3_ is the sonication time. Terms denoted with one asterisk (*) are statistically significant at the 10% level, while those with two asterisks (**) are significant at the 5% level.

Analysis of variance (ANOVA) of the models is shown in Table 2. All models were deemed significant at the 5% level (p<0.012) with insignificant lack of fit (p>0.293). Determination coefficients for all models except PIP were higher than 0.934, indicating very good description of the models. PIP model showed somewhat lower, but still acceptable determination coefficient of 0.698. Adjusted and predicted determination coefficients were in good agreement with each other (within 0.2), while adequate precision of the models was higher than 6, signalling they can be used to navigate the design space.

**Table 2:** ANOVA of the reduced quadratic models used for determination of analytes in processed botanical mixture

Herbal constituent	Model significance (p*-*value)	Lack of fit significance (p*-*value)	PRESS	R^2^	Adjusted R^2^	Predicted R^2^	Adequate precision	*Y*_predicted_/(mg/g)	*Y*_observed_/(mg/g)	Normalized bias/%
green chiretta	<0.001	0.530	4.46	0.934	0.883	0.782	15.97	12.69	12.48	-1.60
black pepper	0.012	0.977	0.18	0.698	0.561	0.482	6.69	1.47	1.41	-3.98
turmeric	<0.001	0.941	0.20	0.965	0.938	0.926	19.09	3.21	3.21	-0.03
Indian frankincense	<0.001	0.293	915.66	0.978	0.964	0.909	24.13	78.63	75.90	-3.47

PRESS=predicted residual sum of squares. Normalized bias is expressed as (observed-predicted)/predicted

**Fig. 2:**
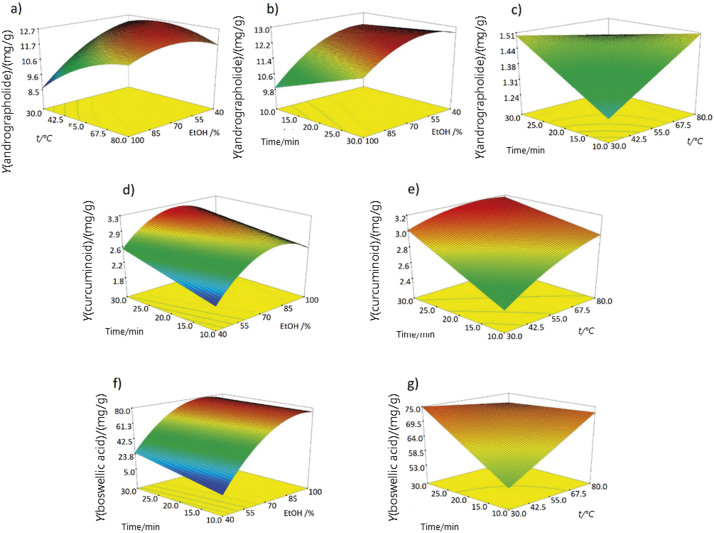
3D response surface plots for: a and b) andrographolide, c) piperine, d and e) curcuminoid, and f and g) boswellic acid yields

Fig. 2 shows the response surface plots for selected factors and responses. The figures confirm the model equations showing that the increase of ethanol ratio in the extraction solvent mostly positively influenced the extraction yields. This is especially evident regarding boswellic acids. Such results are not surprising since the examined analytes are comprised of multiple rings and show moderate to high lipophilicity (log *P* higher than 2.33) (*26*). As for the extraction temperature and time, both factors were positive as linear terms. Their quadratic and interaction terms demonstrated negative influence on the yield, albeit were deemed markedly less significant than the linear terms. This demonstrates the enhancement of extraction with increasing temperature and sonication time, as well as the stability of the analytes during the procedure. The models were used to assess the optimal parameters for maximum yield of active substances. Firstly, each model was considered individually. Then, all models were considered simultaneously to ascertain if there is a considerable difference between the maximum yields predicted individually and simultaneously. Differences between the yields were smaller than 4.02%, so a universal extraction procedure was chosen for all samples in favour of simplicity and high throughput. The predicted optimal extraction parameters were 81.5% ethanol in the extraction solvent, extraction temperature of 60 °C and sonication time of 30 min with overall desirability of 0.927. Lastly, the prediction was validated by extracting the sample in pentaplicate at the predicted optimal parameters. Normalized bias between the observed and predicted yields was less than -3.98%, indicating excellent predictive capability of the model.

To ensure that the extraction solvent does not saturate when analyzing samples with high amounts of analytes, thus leading to falsely low results, solvent-to-sample testing was conducted. Varying amounts of dry extract mixture (10, 50 and 200 mg) were subjected to the extraction procedure in 10 mL of solvent and adequately diluted. A decrease in the determined concentration in each of the tests was not observed, signalling all the analytes were successfully dissolved even at extreme amounts. Therefore, concentrations of 1 mg/mL (as stated in the protocol) can be easily achieved.

### Validation of the methods

#### Selectivity

**Fig. S2:**
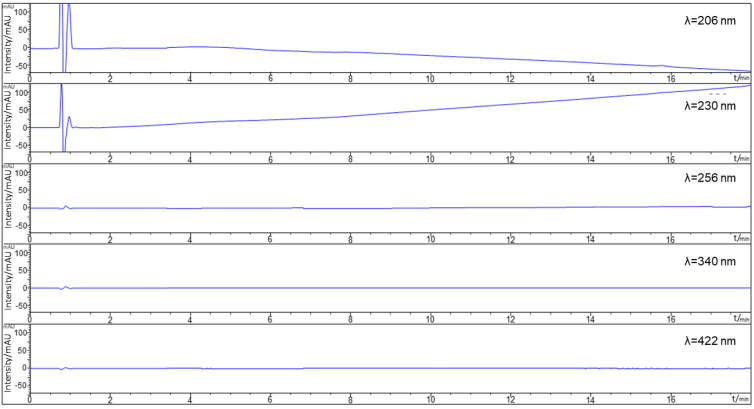
Chromatogram of the excipient solution

Selectivity was examined as the first validation parameter. Firstly, selectivity for extract samples was assessed by analyzing a blend of commonly used excipients (hydroxypropyl methylcellulose, stearic acid, lactose monohydrate, wheat, rice and corn starch and magnesium stearate) mixed in a common ratio present in dosage forms (*27*). As the worst-case scenario, 25 mg of excipient blend were suspended in 25 mL of solvent. The resulting chromatogram is shown in Fig. S2 and reveals no interfering peaks at analyte retention times. Furthermore, peak purity assessment in the range from 200 to 500 nm was conducted on all analyte peaks in standard, processed botanical form and dry extract mixture chromatograms, while the threshold was fixed at 990 (1000 symbolizes a perfectly pure peak). All analytes passed the peak purity tests in the standard chromatogram (peak purity factors were higher than 999.72). In the dry extract and processed botanical form chromatograms, all peaks except BDMC and DMC displayed factors above 997.23, while the two stated did not pass the peak purity test. Coelution of matrix components with BDMC and DMC was evident in chromatograms recorded at lower wavelengths, but it was also observed that the stated matrix components displayed no absorbance at 422 nm, which is used for quantification of curcuminoids. Therefore, the coelution should not present a hindrance for the analysis. As the more reliable method of determining possible interferences in quantification, a standard addition method was employed. The processed botanical mixture was extracted as per the protocol and the extracts were spiked with standard solutions of analytes in the range from 10 to 50 µg/mL (final added concentration). The slopes of the obtained regression lines were then statistically compared to neat standard solution regression line slopes in the same concentration range. The slopes were not statistically different at the significance level of 5% (*t*-test, p>0.073) (Table S2), so it can be concluded that the matrix does not impede the quantification of selected active substances.

#### Linearity, limits of detection and quantification

Linearity was examined using at least five different concentrations in three individual standard solution preparations from which a single regression line was constructed. Linearity was examined in the ranges from LOQ to 200 µg/mL for ANDR, CUR, ABA and BBA (which are more abundant in the samples) and from LOQ to 50 µg/mL for all the other analytes. Table 3 shows the linearity parameters of the method. As can be seen, all lines showed correlation coefficients above 0.999, as well as insignificant y-intercept, indicating satisfactory linearity. The validity of regression lines is further reinforced by the random scatter of datapoints in the residual *vs*. concentration plots. LOD and LOQ were determined using signal-to-noise values of 3 and 10 and were lower than 0.80 and 2.50 µg/mL for all analytes, respectively, as evident in Table 3. No carry-over was observed after the analysis of the highest concentration of the standard solution (all analytes below LOD in the subsequent blank injection).

**Table 3:** Linearity, limit of detection (LOD) and limit of quantification (LOQ) of the proposed method

Analyte	Range *γ*/(µg/mL)	Regression equation	Correlation coefficient	p-value of y intercept	LOD *γ*/(µg/mL)	LOQ *γ*/(µg/mL)
ANDR	0.2–200	y=10.361x+1.092	0.9998	0.841	0.06	0.20
NANDR	1.0–50	y=7.113x+3.911	0.9994	0.102	0.35	1.00
14-DANDR	0.2–50	y=8.054x+0.553	0.9995	0.769	0.05	0.20
PIP	0.1–50	y=33.423x–1.525	0.9993	0.837	0.04	0.10
BDMC	0.1–50	y=29.326x+2.895	0.9998	0.385	0.04	0.10
DMC	0.1–50	y=39.785*x*+6.610	0.9997	0.219	0.03	0.10
CUR	0.1–200	y=39.934x+1.620	0.9990	0.964	0.03	0.10
KBA	0.2–50	y=6.357x+1.494	0.9996	0.150	0.05	0.20
AKBA	0.2–50	y=6.567x+1.385	0.9998	0.092	0.05	0.20
ABA	2.5–200	y=2.666x–0.701	0.9992	0.798	0.80	2.50
BBA	2.5-200	y=2.623x–0.229	0.9995	0.901	0.80	2.50

ANDR=andrographolide, NANDR=neoandrographolide, 14-DANDR=14-deoxy-11,12-didehydroandrographolide, PIP=piperine, BDMC=bisdemethoxycurcumin, DMC=demethoxycurcumin, CUR=curcumin, KBA=11-keto-β-boswellic acid, AKBA=3-*O*-acetyl-11-keto-β-boswellic acid, ABA=α-boswellic acid, BBA=β-boswellic acid

#### Accuracy and precision

Precision of the method was examined by analyzing dry extract and processed botanical mixtures. Repeatability was assessed on six individual sample preparations on the same day, while intermediate precision was examined on six individual preparations over three days. The method proved to be adequately precise, as shown in Table 4; relative standard deviations (RSD) in repeatability examination were lower than 4.17%. As for intermediate precision, yields between the three days did not statistically differ at the significance level of 5% (ANOVA p>0.064). Accuracy of the method was examined by the analysis of standard solutions in triplicate at three different concentration levels (low, medium and high). As shown in Table 4, recoveries varied from 92.3 to 103.4%.

**Table 4:** Measurement of precision and accuracy

Analyte	Processed botanical mixture				Dry extract mixture					*γ*(standard solution)/(μg/mL)		
*γ*(analyte)/(µg/mL)	Repeatability (*N*=6) RSD/%	Intermediate precision (*N*=18)^a^		*γ*(analyte)/(µg/mL)	Repeatability (*N*=6) RSD/%	Intermediate precision (*N*=18)^a^		Low level^b^		Medium level^b^	High level^b^
		*F* (2,15)	p-value			*F* (2,15)	p- value	(Recovery±RSD)/% (*N*=3)			
ANDR	30	2.27	2.638	0.104	200	1.43	1.122	0.351	94.4±2.7		98.4±0.3	95.9±1.0
NANDR	5	3.01	2.474	0.118	10	1.82	3.323	0.064	92.3±4.5		101.4±0.6	99.6±1.2
14-DANDR	15	2.71	2.278	0.137	15	1.96	0.915	0.422	98.9±2.5		103.4±0.4	100.8±1.0
PIP	30	1.31	0.846	0.448	50	2.94	0.131	0.878	97.0±2.7		101.2±0.4	98.5±1.0
BDMC	10	2.50	0.039	0.961	2	4.17	0.498	0.618	91.4±2.8		98.5±0.4	96.5±1.0
DMC	10	0.93	0.743	0.492	10	3.68	0.009	0.991	94.0±2.8		100.7±0.4	98.4±1.0
CUR	20	1.38	1.873	0.188	50	3.68	0.046	0.955	94.1±2.7		98.1±0.4	95.4±0.9
KBA	2	1.23	2.618	0.106	15	0.87	1.162	0.339	94.5±2.6		101.5±0.3	99.4±1.0
AKBA	20	1.25	1.404	0.276	30	0.80	2.033	0.166	96.7±2.7		102.5±0.4	100.1±0.9
ABA	30	1.21	1.101	0.358	50	0.94	1.484	0.158	93.3±3.4		96.7±0.8	94.2±1.1
BBA	120	1.02	2.475	0.118	150	1.41	0.235	0.793	96.9±3.7		102.3±0.5	99.6±1.1

^a^Critical *F* value (*F*_critical_) is equal to 3.682 in all cases, ^b^accuracy of NANDR, 14-DANDR, PIP, BDMC, DMC and AKBA was determined at the concentrations of 5, 25 and 50 µg/mL while the accuracy of ANDR, CUR, ABA and BBA was determined at the concentrations of 20, 100 and 200 µg/mL. RSD=relative standard deviation, ANDR=andrographolide, NANDR=neoandrographolide, 14-DANDR=14-deoxy-11,12-didehydroandrographolide, PIP=piperine, BDMC=bisdemethoxycurcumin, DMC=demethoxycurcumin, CUR=curcumin, KBA=11-keto-β-boswellic acid, AKBA=3-*O*-acetyl-11-keto-β-boswellic acid, ABA=α-boswellic acid, BBA=β-boswellic acid

#### Stability

Stability of the analytes was examined during the extraction procedure and in the autosampler. Firstly, to ensure no analyte degradation occurs during the extraction process, a standard solution was prepared by light vortexing, analyzed, subjected to the extraction procedure and analyzed again. There was no observed decrease in the concentration after sonication (peak area decrease was lower than 1%), which shows stability of the analytes as the Box-Behnken models have already implied. Afterwards, autosampler stability was assessed for the standard, dry extract mixture and processed botanical mixture solutions kept in the autosampler at 15 °C for up to three days. The decrease of peak area within one day was less than 0.95, 1.83 and 4.79% for standards, dry extract and processed botanical form, respectively. It was concluded that the samples are stable up to a day, in case of prolonged analyses.

#### Robustness

Robustness was evaluated using a Plackett-Burman design (Design Expert v. 7.0.0 software (*25*)). Effect of sonication time (A, in min), extraction temperature (C, in °C), ethanol ratio in the extraction solvent (E, in %), mobile phase flow rate (G, in mL/min), column temperature (H, in °C), detection wavelength change (J, in nm) and gradient change (K, in percentage of mobile phase component B) on analyte yield (mg/g of mixture) and resolution was examined. Testing was conducted on a representative processed botanical mixture. Experiment design and the obtained responses for each run are shown in Table S3 and Table S4, respectively. The obtained models were analyzed and the factor effects for each model were determined. Critical effects for a response were also estimated at 5 and 1% levels using standard errors of the effect and tabulated *t*-values. The results are shown in Table S5. Significant and important effects according to the comparison of critical and negligible effects, Pareto chart and half-normal probability plot were calculated. The results are shown in Table S6. As it can be seen, sample preparation conditions (extraction time and temperature, and ethanol ratio) influence a small number of responses (for example, only BDMC yield and resolution from comparison with critical and negligible effects at a significance level of 5%, none at 1%). As these are the factors most prone to human error, the method can be considered robust in this aspect. Finally, insignificant intervals for each significant factor were determined from critical and factor effects. The results are shown in Table S7. Flow rate, column temperature, gradient change and detection wavelength are factors most commonly influencing the responses. In addition, their insignificant intervals are relatively narrow, which implies that a robust chromatographic system in terms of temperature, flow and gradient control is a necessity.

### Identification of acetylated boswellic acids

**Fig. S3:**
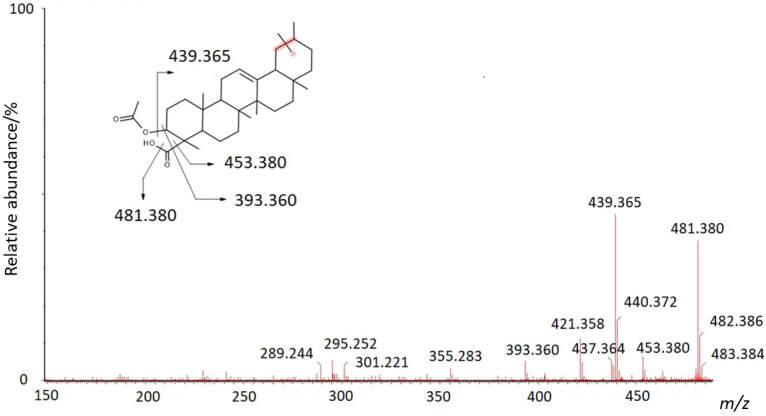
Representative MS/MS spectrum (precursor *m*/*z*=499.4) of a peak at 15.32 min. Inset: proposed fragmentation pattern of acetylated boswellic acids

As six boswellic acids constitute the majority of Indian frankincense resin and the acid content of its extracts, quantification of only four of them could lead to falsely low results. Being unable to procure two boswellic acid standards (AABA and ABBA), we decided to quantify them using calibration curves of ABA and BBA, respectively, since the literature search revealed similar calibration slopes of acetylated and non-acetylated forms using spectrophotometric detection at 200–210 nm (*28*, *29*). Mass spectrometry was used to identify AABA and ABBA peaks in the frankincense processed botanical form sample. MS scans revealed that peaks at 14.84 and 15.32 min contain peak *m/z=*499.385, which are likely quasi-molecular ions of the analytes of interest. Monoisotopic masses of AABA and ABBA after the addition of a proton (*m/z=*499.379) are in high accordance with this value. MS/MS analyses were further carried out with the peak *m/z=*499.4 as the precursor. Peaks at 14.84 and 15.32 min showed the same fragmentation pattern with fragment ions at *m/z*=481.380 (loss of hydroxyl moiety of the carboxyl group), 453.380 (cleavage of the carboxyl group), 439.365 (cleavage of the acetoxy group) and 393.360 (loss of both acetoxy and carboxyl groups) (Fig. S3). The combination of chromatographic behaviour, relative abundance in the samples compared to other boswellic acids, UV-Vis spectra and mass spectra all imply that the peaks at 14.84 and 15.32 min are AABA and ABBA, respectively.

### Sample analysis

Analysis of the samples revealed large differences in the content of active substances (Table 5), as well as discrepancies between the found and declared contents (Table 6).

**Table 5:** Results of the mass uniformity and mass fraction analyses of dosage forms

Sample	*m*(dosage)_variation_ RSD/%, *N*=6	*w*(analyte)/(mg/g)												
ANDR	NANDR	14-DANDR	PIP	BDMC	DMC	CUR	KBA	AKBA	ABA	BBA	AABA	ABBA
S1	1.90	n/e	n/e	n/e	23.9±5.7	0.8±4.2	1.0±6.4	231.7±3.8	<LOQ	<LOQ	<LOQ	<LOQ	<LOQ	<LOQ
S2	2.68	n/e	n/e	n/e	12.1±1.2	99.6±3.3	147.8±2.8	590.6±2.8	n/e	n/e	n/e	n/e	n/e	n/e
S3	0.74	n/e	n/e	n/e	0.4±11.9	0.7±3.9	3.4±0.4	16.6±1.2	n/e	n/e	n/e	n/e	n/e	n/e
S4	2.86	n/e	n/e	n/e	n/e	23.1±1.7	151.3±1.9	695.9±2.0	n/e	n/e	n/e	n/e	n/e	n/e
S5	3.84	n/e	n/e	n/e	n/e	7.7±2.2	55.8±3.7	266.2±3.6	2.2±4.3	61.2±5.1	10.3±8.5	32.6±7.6	17.6±2.3	169.7±8.3
S6	1.09	n/e	n/e	n/e	<LOD	7.4±2.2	4.8±1.0	12.1±0.6	n/e	n/e	n/e	n/e	n/e	n/e
S7	1.76	n/e	n/e	n/e	n/e	0.3±2.3	4.9±0.5	51.6±0.2	n/e	n/e	n/e	n/e	n/e	n/e
S8	2.97	n/e	n/e	n/e	n/e	20.0±4.0	109.3±3.3	581.2±3.1	n/e	n/e	n/e	n/e	n/e	n/e
S9	2.89	n/e	n/e	n/e	9.1±3.0	34.1±1.7	142.5±1.7	717.4±1.8	n/e	n/e	n/e	n/e	n/e	n/e
S10	0.78	n/e	n/e	n/e	n/e	2.2±2.4	10.0±0.8	46.6±0.6	n/e	n/e	n/e	n/e	n/e	n/e
S11	2.82	n/e	n/e	n/e	9.3±4.3	23.9±1.9	131.6±2.3	684.1±2.5	n/e	n/e	n/e	n/e	n/e	n/e
S12	2.22	n/e	n/e	n/e	3.0±2.6	1.8±3.5	8.4±2.8	43.2±2.1	1.4±20.7	1.2±37.9	5.3±2.7	12.5±4.5	<LOQ	3.0±5.6
S13	5.41	n/e	n/e	n/e	0.9±9.6	7.6±1.8	5.9±0.7	14.3±0.7	n/e	n/e	n/e	n/e	n/e	n/e
S14	/	n/e	n/e	n/e	7.8±8.2	4.7±2.5	18.5±3.4	229.5±4.2	39.9±3.3	29.8±3.3	129.0±3.5	267.9±2.4	24.8±3.3	60.8±2.8
S15	/	n/e	n/e	n/e	n/e	0.2±1.2	0.1±0.5	0.1±0.4	n/e	n/e	n/e	n/e	n/e	n/e
S16	/	n/e	n/e	n/e	n/e	0.1±3.6	<LOQ	<LOQ	n/e	n/e	n/e	n/e	n/e	n/e
S17	0.45	n/e	n/e	n/e	n/e	1.9±1.1	11.5±0.2	48.7±0.3	n/e	n/e	n/e	n/e	n/e	n/e
S18	1.37	n/e	n/e	n/e	n/e	0.3±7.3	1.9±1.9	11.7±2.2	n/e	n/e	n/e	n/e	n/e	n/e
S19	1.18	n/e	n/e	n/e	6.2±8.7	16.3±0.5	76.5±1.0	379.8±1.0	n/e	n/e	n/e	n/e	n/e	n/e
S20	0.99	n/e	n/e	n/e	n/e	<LOQ	0.3±3.0	1.7±1.8	<LOQ	<LOQ	0.9±4.6	2.2±2.8	<LOQ	<LOQ
S21	/	n/e	n/e	n/e	190.2±2.8	n/e	n/e	n/e	n/e	n/e	n/e	n/e	n/e	n/e
S22	/	n/e	n/e	n/e	n/e	n/e	n/e	n/e	42.8±2.7	32.4±2.8	135.7±4.7	299.4±1.7	18.0±9.3	74.9±2.9
S23	5.75	n/e	n/e	n/e	n/e	n/e	n/e	n/e	17.6±1.3	15.7±1.0	53.4±2.3	121.4±0.6	9.8±2.2	33.6±1.2
S24	2.94	n/e	n/e	n/e	n/e	n/e	n/e	n/e	35.0±1.5	12.9±1.7	97.5±0.9	196.2±1.5	11.4±4.3	34.9±3.7
S25	3.20	n/e	n/e	n/e	n/e	n/e	n/e	n/e	0.7±6.8	2.3±4.3	2.4±6.9	5.0±3.1	<LOQ	2.5±5.0
S26	1.08	n/e	n/e	n/e	n/e	n/e	n/e	n/e	44.0±0.8	84.3±0.5	131.2±0.9	294.8±0.9	26.2±2.4	56.2±1.1
S27	2.54	n/e	n/e	n/e	n/e	n/e	n/e	n/e	18.5±10.1	7.2±10.2	67.6±11.0	151.7±10.6	13.3±10.0	25.4±11.3
S28	1.29	n/e	n/e	n/e	n/e	n/e	n/e	n/e	0.1±10.6	0.2±1.1	0.3±15.3	0.7±12.4	0.1±19.1	0.2±19.7
S29	1.21	n/e	n/e	n/e	n/e	n/e	n/e	n/e	0.8±0.3	7.6±0.3	2.5±0.8	5.3±0.7	0.4±2.8	1.2±0.5
S30	/	912.0±1.4	<LOD	<LOQ	n/e	n/e	n/e	n/e	n/e	n/e	n/e	n/e	n/e	n/e
S31	3.08	146.7±2.8	5.1±0.7	8.0±0.6	n/e	n/e	n/e	n/e	n/e	n/e	n/e	n/e	n/e	n/e
S32	2.73	<LOD	<LOD	<LOD	n/e	n/e	n/e	n/e	n/e	n/e	n/e	n/e	n/e	n/e
S33	1.37	6.9±3.7	<LOD	0.1±1.8	<LOD	<LOD	<LOD	<LOD	0.8±1.2	0.7±1.1	2.0±1.7	5.0±1.2	0.3±9.0	1.5±2.7
S34	1.68	149.1±6.0	3.8±10.0	6.7±3.7	n/e	10.3±5.9	37.1±5.8	174.8±5.6	n/e	n/e	n/e	n/e	n/e	n/e
S35	0.90	0.6±9.2	<LOD	<LOD	n/e	6.0±5.2	8.5±3.6	34.4±3.4	n/e	n/e	n/e	n/e	n/e	n/e
S36	1.20	n/e	n/e	n/e	n/e	2.0±7.9	6.3±8.5	28.8±7.7	n/e	n/e	n/e	n/e	n/e	n/e
S37	/	n/e	n/e	n/e	n/e	9.8±2.1	9.1±1.2	24.0±1.0	n/e	n/e	n/e	n/e	n/e	n/e
S38	/	n/e	n/e	n/e	n/e	9.8±1.8	8.0±0.6	21.8±0.4	n/e	n/e	n/e	n/e	n/e	n/e
S39	/	n/e	n/e	n/e	n/e	11.5±1.9	10.0±1.4	23.9±1.0	n/e	n/e	n/e	n/e	n/e	n/e
S40	/	n/e	n/e	n/e	n/e	10.6±1.9	6.6±1.3	15.9±1.4	n/e	n/e	n/e	n/e	n/e	n/e
S41	1.28	n/e	n/e	n/e	3.4±1.7	4.2±4.8	3.7±1.9	10.2±1.3	n/e	n/e	n/e	n/e	n/e	n/e
S42	3.77	n/e	n/e	n/e	2.8±4.7	27.6±0.5	15.1±0.3	36.8±0.2	n/e	n/e	n/e	n/e	n/e	n/e
S43	5.31	n/e	n/e	n/e	n/e	40.3±0.5	18.6±0.3	43.4±0.3	n/e	n/e	n/e	n/e	n/e	n/e
S44	/	n/e	n/e	n/e	3.8±13.9	4.0±3.3	3.0±4.8	7.7±4.8	n/e	n/e	n/e	n/e	n/e	n/e
S45	/	n/e	n/e	n/e	n/e	8.0±1.0	6.4±0.6	17.4±0.7	n/e	n/e	n/e	n/e	n/e	n/e
S46	/	n/e	n/e	n/e	n/e	7.6±1.5	5.7±0.1	14.1±0.4	n/e	n/e	n/e	n/e	n/e	n/e
S47	4.27	n/e	n/e	n/e	1.2±3.6	13.9±1.0	6.3±1.1	12.4±1.5	n/e	n/e	n/e	n/e	n/e	n/e
S48	/	n/e	n/e	n/e	n/e	15.0±2.8	11.3±2.2	27.4±2.2	n/e	n/e	n/e	n/e	n/e	n/e
S49	/	n/e	n/e	n/e	n/e	n/e	n/e	n/e	11.1±5.9	21.5±1.7	52.3±2.9	150.3±2.6	24.6±5.7	45.5±5.8
S50	/	n/e	n/e	n/e	n/e	n/e	n/e	n/e	6.7±4.5	40.2±4.6	31.7±3.0	125.4±3.2	25.2±2.0	50.5±4.6
S51	/	n/e	n/e	n/e	n/e	n/e	n/e	n/e	4.1±2.0	24.3±0.9	35.9±1.8	126.7±1.1	22.7±2.9	63.2±0.2
S52	/	14.5±6.5	2.3±10.0	6.1±7.1	n/e	n/e	n/e	n/e	n/e	n/e	n/e	n/e	n/e	n/e
S53	/	9.4±0.7	1.1±8.6	1.6±0.5	n/e	n/e	n/e	n/e	n/e	n/e	n/e	n/e	n/e	n/e
S54	3.87	33.9±0.4	6.5±1.4	4.0±0.7	n/e	n/e	n/e	n/e	n/e	n/e	n/e	n/e	n/e	n/e

A detailed description of all analyzed samples (S1-S54), including mode of acquisition, manufacturer origin, sample type and label, is given in Table S1. Results for mass fraction of analyte in dosage are mean value±RSD, *N*=3. RSD=relative standard deviation. n/e=not expected, ANDR=andrographolide, NANDR=neoandrographolide, 14-DANDR=14-deoxy-11,12-didehydroandrographolide, PIP=piperine, BDMC=bisdemethoxycurcumin, DMC=demethoxycurcumin, CUR=curcumin, KBA=11-keto-β-boswellic acid, AKBA=3-*O*-acetyl-11-keto-β-boswellic acid, ABA=α-boswellic acid, BBA=β-boswellic acid, AABA=3-*O*-acetyl-α-boswellic acid, ABBA=3-*O*-acetyl-β-boswellic acid. LOQ=below the limit of quantification, LOD=below the limit of detection

**Table 6:** Found/declared mass ratio of herbal component in dry extract and botanical processed samples, and daily intake of active substances

Sample	*m*(sample)/mg	(*m*(substance)_found_/*m*(substance)_declared_)/%				*m*(daily intake)/mg^a^			
Andrographolide	Piperine	Curcuminoid	Boswellic acid	Andrographolide	Piperine	Curcuminoid	Boswellic acid
S1	456	n/e	n/d	n/d	n/d	n/e	21.8	213.0	<LOQ
S2	403	n/e	102.7	101.6	n/e	n/e	9.8	675.4	n/e
S3	1169	n/e	5.0	50.4	n/e	n/e	0.5	24.2	n/e
S4	501	n/e	n/e	91.8	n/e	n/e	n/e	872.0	n/e
S5	507	n/e	n/e	70.3	79.4 (AKBA 124.1)	n/e	n/e	334.3	297.7
S6	293	n/e	<LOQ	2.8	n/e	n/e	<LOQ	28.5	n/e
S7	694	n/e	n/e	93.9	n/e	n/e	n/e	315.4	n/e
S8	486	n/e	n/e	90.9	n/e	n/e	n/e	690.6	n/e
S9	541	n/e	98.4	96.7	n/e	n/e	4.9	483.7	n/e
S10	886	n/e	n/e	108.5	n/e	n/e	n/e	52.1	n/e
S11	486	n/e	95.4	95.2	n/e	n/e	9.0	816.1	n/e
S12	853	n/e	90.3	95.9	66.5 (AKBA 11.4)	n/e	5.1	91.1	40.0
S13	322	n/e	6.1	1.9	n/e	n/e	1.7	53.7	n/e
S14	/	n/e	41.0	53.2	176.7	n/e	6.4	202.2	441.8
S15	/	n/e	n/e	n/d	n/e	n/e	n/e	0.5	n/e
S16	/	n/e	n/e	1.4	n/e	n/e	n/e	0.1	n/e
S17	452	n/e	n/e	93.6	n/e	n/e	n/e	28.1	n/e
S18	1600	n/e	n/e	95.7	n/e	n/e	n/e	44.5	n/e
S19	340	n/e	n/d	102.3	n/e	n/e	6.3	482.1	n/e
S20	1623	n/e	n/e	n/d	n/d	n/e	n/e	6.6	7.8
S21	/	n/e	20.0	n/e	n/e	n/e	4.0	n/e	n/e
S22	/	n/e	n/e	n/e	92.8	n/e	n/e	n/e	482.6
S23	291	n/e	n/e	n/e	38.6	n/e	n/e	n/e	146.4
S24	441	n/e	n/e	n/e	60.0	n/e	n/e	n/e	342.1
S25	482	n/e	n/e	n/e	2.0	n/e	n/e	n/e	12.4
S26	314	n/e	n/e	n/e	70.7	n/e	n/e	n/e	399.8
S27	391	n/e	n/e	n/e	119.6^b^	n/e	n/e	n/e	110.9
S28	1073	n/e	n/e	n/e	8.6	n/e	n/e	n/e	3.4
S29	1647	n/e	n/e	n/e	90.2^b^	n/e	n/e	n/e	58.6
S30	/	93.1	n/e	n/e	n/e	91.2	n/e	n/e	n/e
S31	391	n/d	n/e	n/e	n/e	125.0	n/e	n/e	n/e
S32	504	n/d	n/e	n/e	n/e	<LOQ	n/e	n/e	n/e
S33	694	n/d	n/d	n/d	n/d	14.6	<LOQ	<LOQ	21.4
S34	528	n/d	n/e	117.2	n/e	168.5	n/e	234.6	n/e
S35	880	n/d	n/e	107.9	n/e	3.2	n/e	258.2	n/e
S36	699	n/e	n/e	n/d	n/e	n/e	n/e	26.0	n/e
S37	/	n/e	n/e	4.3	n/e	n/e	n/e	n/a	n/e
S38	/	n/e	n/e	4.0	n/e	n/e	n/e	n/a	n/e
S39	/	n/e	n/e	4.5	n/e	n/e	n/e	n/a	n/e
S40	/	n/e	n/e	3.3	n/e	n/e	n/e	n/a	n/e
S41	400	n/e	5.2	3.5	n/e	n/e	2.7	14.5	n/e
S42	483	n/e	2.8	8.8	n/e	n/e	8.1	230.4	n/e
S43	395	n/e	n/e	10.2	n/e	n/e	n/e	40.4	n/e
S44	/	n/e	7.6	3.0	n/e	n/e	n/a	n/a	n/e
S45	/	n/e	n/e	3.2	n/e	n/e	n/e	n/a	n/e
S46	/	n/e	n/e	2.7	n/e	n/e	n/e	n/a	n/e
S47	297	n/e	4.4	4.0	n/e	n/e	3.6	96.8	n/e
S48	/	n/e	n/e	5.4	n/e	n/e	n/e	n/a	n/e
S49	/	n/e	n/e	n/e	30.5	n/e	n/e	n/e	n/a
S50	/	n/e	n/e	n/e	28.0	n/e	n/e	n/e	n/a
S51	/	n/e	n/e	n/e	27.8	n/e	n/e	n/e	n/a
S52	/	2.3	n/e	n/e	n/e	22.9	n/e	n/e	n/e
S53	/	1.2	n/e	n/e	n/e	48.4	n/e	n/e	n/e
S54	425	4.4	n/e	n/e	n/e	301.9	n/e	n/e	n/e

A detailed description of all analyzed samples (S1-S54), including mode of acquisition, manufacturer origin, sample type and label, is given in Table S1. *m*(sample)=mean value of *N*=6, ^a^per dosing regimen recommended by the manufacturer, ^b^assuming *w*(boswellic acid)=65% in the extract, n/e=not expected, n/d=content not declared, LOQ=below the limit of quantification, n/a=recommended daily intake not stated. AKBA=3-*O*-acetyl-11-keto-β-boswellic acid, LOQ=below the limit of quantification

Firstly, in the extract samples S1-S35, the mass fraction of curcuminoids varied the most, ranging from 0.1 (S16) to 894 mg/g of sample (S9). USP monograph (*10*) prescribes a certain curcuminoid distribution for turmeric extracts, *viz*. 70-80% CUR, 15-25% DMC and 2.5-6.5% BDMC. Seven of mainly turmeric-based preparations did not satisfy this criterion. Among the most striking ones are S1, which contains above 99% CUR, and S6 and S13, in which the relative content of BDMC is 30%. These results point to possible adulteration with synthetic CUR (S1 and S14) and adulteration with synthetic BDMC or *Curcuma* species other than *C. longa* (S6, S13), raising concerns (*30*). Mass fraction of PIP ranged from undetectable to a high 190.2 mg/g. Andrographolide mass fraction in sample S30 was estimated at 912.0 mg/g, although it consisted of only ANDR and virtually no other andrographolides, which could imply adulteration with the pure substance. Samples S31, S33 and S34 contained markedly lower mass fractions of andrographolides (below 159.8 mg/g), but with a profile more characteristic of the herbal constituent. Sample S32, even though claiming to contain both the processed botanical form and extract in appreciable amounts, showed no andrographolides above the detection limit, while sample S35 contained trace amounts of andrographolides (0.6 mg/g). Indian frankincense-based samples contained from 1.6 (S28) to 636.7 (S26) mg/g of boswellic acids. Most pharmacologically active keto derivatives were in the range from below LOQ to 128.3 mg/g (S26). Interestingly, one product (S25), purchased from an online supplier, claiming to be pure frankincense extract standardized to 65% boswellic acids only displayed 12.9 mg/g of all six boswellic acids and a miniscule 3.0 mg/g of keto derivatives. As for the found *vs*. declared content, about half of the turmeric-containing samples with stated content (12 of 19) conformed to the USP criteria of 90 to 110% declared content. A few samples (namely S6, S13 and S16) showed contents lower than 20% declared. It should be pointed out that these products were purchased from an online retailer. A recent survey of products on the American market revealed good agreement of declared and found content in the products from local suppliers, similar to the findings for the locally bought products in Croatia (*30*), which points to the questionable quality of products bought from dubious Internet sources. PIP content conformed to the product declaration in 4 out of 9 samples. Samples containing equal to or less than 20% declared content (S3, S13 and S21) can be presumed to be underdosed in recommended dosing regimens, especially sample S21, which consists of only black pepper extract. Sample S30 was the only green chiretta sample to have unambiguously declared content, also conforming to the USP requirements (93.1% of declared content). As for the Indian frankincense samples, USP criteria could not be applied here since most manufacturers do not declare the content of keto derivatives, only total boswellic acids. The content of boswellic acids spanned from 2.0 (S25) to 176.7% (S14) of the declared values, although the extracts are standardized to minimum rather than absolute content of boswellic acids. Two of 11 products contained more than 90% of declared boswellic acids, while only one product exhibited KBA below 1% of total boswellic acids (S5); however, it contained elevated amounts of AKBA, which is the more potent component.

Materials such as herbal substances and their processed forms can also be taken as dietary supplements or be used as raw material in the manufacture of various preparations. Therefore, the content of active substances in the processed botanical forms (S36-S54) is also of paramount importance for the quality of the final product. Active substance content is again expressed as yield (mg/g of sample) (Table 5), as well as a mass ratio with regard to the mass of the corresponding herbal component in the sample (Table 6). Content of curcuminoids varied from 14.7 to 102.3 mg/g of sample, while the mass fractions varied from 2.7 to 10.2%. Similar results have been obtained by other research groups (*31*, *32*). Ph. Eur. (*11*) prescribes 2% curcuminoids as the lowest limit, which all samples satisfy. Only one sample did not conform to the USP criterion of 3%, albeit barely (2.7%, S46). Two of the samples (S42 and S43) contained an unusually high content of curcuminoids (8.8 and 10.2%, respectively). Upon examining the curcuminoid profile, amounts of BDMC comparable to CUR are observable, which is not typical of *C. longa* and could point to substitution or adulteration. Green chiretta samples contained from 12.1 (S53) to 44.4 mg/g (S54) of andrographolides (1.2 to 4.4%), conforming to the Ph. Eur. and USP limits of 0.8 and 1%, respectively. Sample S54, from Thailand, contained the most andrographolides, which is consistent with the higher amounts found on the Thai market (*33*). Frankincense resin contained 27.8 to 30.5% boswellic acids, complying with the literature (*34*). All samples containing above 1.5% KBA and 7.0% AKBA conform to both Ph. Eur. and USP (*10*, *11*) criteria for keto derivatives.

The estimated daily intake of each active substance group per sample is shown in Table 6. Drastic differences in the highest and lowest estimated intakes of active substance groups between different samples can be observed (up to 9000 times, curcuminoids in S4 and S16). As is expected, active substance intake in the processed botanical form samples is lower than in extracts (the exception being andrographolides in S54, 301.9 mg). As for the preparations, few demonstrated drastically low intakes of curcuminoids without any adjuvants (S6, S15, S16 and S20, below 30 mg/day). A few samples also demonstrated lower values, although they were coupled with PIP or formulated in lipid vesicles, which enhances the bioavailability of curcuminoids. Samples S20, S25, S28 and S33 showed very low content of boswellic acids (estimated daily intake lower than 12.4 mg/day). Apart from S20, S25 and S33, which are combination products, it can be presumed that sample S25 has little to no therapeutic value. On the other hand, high daily intakes were also observed in multiple products (up to 872.0 mg of curcuminoids in S4, 21.8 mg of PIP in S1, 482.6 mg of boswellic acids in S22 and 301.9 mg of andrographolides in S54). Curcuminoids are shown to be safe up to 12 g a day in healthy individuals, while doses of around 500-1000 mg daily (or less with absorption enhancement) demonstrated therapeutic efficacy in various inflammatory diseases (*35*). Boswellic acids improved inflammatory bowel disease and arthritis symptoms in doses of 100–500 mg and higher with a good safety profile, even more so when AKBA was present in higher amounts (*5*, *32*), which would imply that S5 and S26 were the most desirable in terms of boswellic acid content. However, daily intake of andrographolides and PIP when using products S54 and S1, respectively, raises concerns. Although andrographolides show good safety and efficacy in doses up to 100 mg/day, studies have shown doses of 5 mg/(kg·day) and higher can cause various side-effects such as allergic reactions, diarrhoea, heartburn, *etc*. (*36*). In addition, *in vitro* and *ex vivo* studies have suggested that 14-DANDR could have a hypotensive effect in higher doses (*37*). An intake of 301.9 mg of andrographolides daily using S54 could, thus, lead to possible adverse effects. As for PIP, doses of 1–10 mg daily are sufficient for bioavailability enhancement; however, simultaneous use of doses higher than 10 mg with CYP3A or P-glycoprotein substrates could affect their pharmacokinetics, leading to impaired safety and efficacy (*38*). As supplementation with S1 can provide a single dose of 21.8 mg PIP, there is a potential for interaction with concomitantly used drugs. Although there are no exact guidelines for maximum dosages of these substances, care should be taken with older patients on polytherapy or with liver disease.

## CONCLUSIONS

The method for determination of three andrographolides, three curcuminoids, six boswellic acids and piperin has been successfully developed and validated for the first time in food and dietary supplement samples. The method is fast, accurate and precise, with simple sample preparation and can be used for various types of samples, especially combination products of stated species which are more and more prevalent today. This study has its limitations, mainly regarding Indian frankincense-based samples. Firstly, the content of 3-*O*-acetyl­α- and β-boswellic acids (AABA and ABBA) was obtained using calibration curves of ABA and BBA, respectively, which introduces an error due to different molar absorption coefficients of the analytes. Secondly, a resolution of 1.5 could not be achieved between ABA and BBA, as well as among AABA, ABBA and matrix components without severely prolonging the method and compromising throughput. However, both these obstacles are not expected to alter the results significantly and the method can be used as an adequate estimate of sample quality in terms of boswellic acid content, especially since the keto derivatives are presumed to exert the most potent pharmacological effect. For this reason, the manufacturers should also state the content of keto derivatives in the extract samples (as the USP demands), which only a fraction have done. Regarding content analysis, products bought from online suppliers were shown to either drastically deviate from the declared content, contain little to no active substances (implying no therapeutic effect), or contain large amounts which could lead to adverse effects when taking the supplement as recommended. Products bought this way can bypass certain food and dietary supplement regulatory requirements, being directly delivered to the consumer, thus endangering their well-being. In conclusion, consumers should refrain from buying food and supplements from the Internet and instead procure them from certified pharmacies and food health stores.

## Appendix

**Table S1:** List of analyzed samples

Code	Purchased from	Manufacturer location	Matrix	Sample type	Description/label (per dosage form, unless specified otherwise)
S1	online supplier	Kakheri, India	dry extract	hard capsules	turmeric (standardized to min. 90% curcuminoids), Indian frankincense (standardized to min. 65% boswellic acids), black pepper fruit (standardized to min. 95% piperine)
S2	local pharmacy	Zagreb, Croatia	dry extract	hard capsules	turmeric extract (350 mg, standardized to 9% curcuminoids), black pepper extract (5 mg, standardized to 95% piperine)
S3	local pharmacy	Zagreb, Croatia	soft extract	soft capsules	turmeric extract (containing 48 mg of curcuminoids, of which 40 mg curcumin), black pepper extract (10 mg), vitamin D_3_ (3 µg)
S4	local health store	Karlovac, Croatia	dry extract	hard capsules	mixture of turmeric dry extract and turmeric root oil (25:1) (standardized to 95% curcuminoids)
S5	local pharmacy	Karlovac, Croatia	dry extract	hard capsules	mixture of turmeric dry extract and turmeric root oil (25:1) (250 mg, standardized to 95% curcuminoids), Indian frankincense extract (250 mg, standardized to 10% AKB and 75% boswellic acids)
S6	online supplier	Loei, Thailand	dry extract	tablets	turmeric extract/black pepper extract (10:1)
S7	local pharmacy	Baillonville, Belgium	soft extract	soft capsules	bio-optimized turmeric extract (standardized to 42 mg curcumin/capsule)
S8	local pharmacy	Milan, Italy	dry extract	hard capsules	turmeric extract (standardized to 95% curcuminoids)
S9	local pharmacy	Salzburg, Austria	dry extract	hard capsules	turmeric extract (528 mg, of which 500 mg curcuminoids), black pepper fruit extract (5.3 mg, of which 5 mg piperine)
S10	local pharmacy	Leonia, NJ, USA	soft extract	soft capsules	turmeric extract (48 mg curcuminoids, of which 40 mg curcumin/capsule)
S11	local pharmacy	Zagreb, Croatia	dry extract	hard capsules	turmeric rhizome dry extract (450 mg, standardized to min. 95% curcuminoids), black pepper fruit dry extract (10 mg, standardized to min. 95% piperine), vitamin B_6_ (0.7 mg), vitamin B_12_ (1.25 µg), vitamin D_3_ (2.5 µg), selenium (27.5 µg)
S12	local pharmacy	Zagreb, Croatia	dry extract	hard capsules	turmeric rhizome dry extract (50 mg, standardized to min. 95% curcuminoids), vitamin C (50 mg), frankincense resin dry extract (40 mg, standardized to min. 75% boswellic acid, of which min. 30% AKBA), hyaluronic acid (15 mg), manganese (1 mg), black pepper dry extract (3 mg, standardized to min. 95% piperine), vitamin D_3_ (2.5 µg)
S13	online supplier	not stated	dry extract	hard capsules	turmeric root extract powder (500 mg, standardized to 95% curcuminoids), BioPerine black pepper extract powder (5 mg)
S14	online supplier	Sonipat, India	dry extract	powder bulk	curcumin (200 mg, standardized to 95% curcuminoids), frankincense extract (192 mg, standardized to 65% boswellic acids), black pepper extract (8 mg, standardized to 95% piperine)
S15	local health store	Zagreb, Croatia	tincture	liquid	eco turmeric
S16	online supplier	Ruen, Bulgaria	tincture	liquid	turmeric (900 mg/daily dose)
S17	local pharmacy	Zagreb, Croatia	soft extract	soft capsules	curcumin (30 mg), vitamin D_3_ (7 µg)
S18	local health store	Leonia, NJ, USA	dry extract	tablets	turmeric root extract (25 mg, standardized to 93% curcuminoids), vitamin B, vitamin C, vitamin E, iron, broccoli extract, tea extract
S19	local health store	Dorset, UK	dry extract	tablets	turmeric extract (175 mg, standardized to 95% curcuminoids)
S20	online supplier	Park City, UT, USA	dry extract	tablets	Indian frankincense resin extract, turmeric root extract, glucosamine, chondroitin, methylsulfonylmethane, phenylalanine, bromelain, calcium, zinc, manganese, boron
S21	online supplier	Sonipat, India	dry extract	powder bulk	standardized to min. 95% piperine
S22	online supplier	Sonipat, India	dry extract	powder bulk	min. 60% boswellic acids
S23	online supplier	Sonipat, India	dry extract	hard capsules	Indian frankincense resin extract (500 mg, standardized to min. 65% boswellic acids)
S24	online supplier	Rohtak, India	dry extract	hard capsules	Indian frankincense resin extract (500 mg, standardized to min. 75% boswellic acids)
S25	online supplier	London, UK	dry extract	hard capsules	Indian frankincense resin extract (482 mg, standardized to min. 65% boswellic acids)
S26	online supplier	Rohtak, India	dry extract	hard capsules	Indian frankincense resin extract (500 mg, standardized to min. 90% boswellic acids)
S27	local health store	Samobor, Croatia	dry extract	hard capsules	Indian frankincense resin extract (140 mg), balmtree extract (140 mg), bovine colostrum (70 mg)
S28	local pharmacy	Zagreb, Croatia	dry extract	hard capsules	Indian frankincense resin extract (80 mg, min. 25% boswellic acids), glucosamine, chondroitin, vitamin C, hyaluronic acid
S29	local pharmacy	Boca Raton, FL, USA	dry extract	tablets	5-LOXIN advanced AKBA (50 mg), glucosamine, vitamin C, manganese, boron, hyaluronic acid
S30	online supplier	Rohtak, India	dry extract	hard capsules	standardized to min. 98% andrographolides
S31	online supplier	Rohtak, India	dry extract	hard capsules	green chiretta extract (50 mg, min. 90% andrographolides), powdered green chiretta herb (350 mg)
S32	online supplier	Indore, India	dry extract	hard capsules	green chiretta extract (600 mg, min. 2% andrographolides), powdered green chiretta herb (200 mg)
S33	online supplier	Hollywood, FL, USA	dry extract	hard capsules	proprietary blend (turmeric, green chiretta, Indian frankincense extracts, piperine, glucosamine, collagen, hyaluronic acid)
S34	online supplier	Fort Lauderdale, FL, USA	dry extract	hard capsules	turmeric extract (250 mg, standardized to 40% curcuminoids), PARACTIN® (150 mg, patented blend of andrographolides)
S35	online supplier	Tampa, FL, USA	dry extract	hard capsules	green chiretta herb (25 mg), turmeric root extract (42 mg, standardized to 95% curcuminoids), vitamins C and D, magnesium, selenium, zinc, elderberry fruit extract, olive leaf extract, kudzu root powder, N-acetylcysteine, garlic bulb powder, quercetin, oregano leaf powder
S36	local pharmacy	Toronto, Canada	dry extract + processed botanical form	hard capsules	turmeric rhizome (500 mg), turmeric rhizome extract (50 mg, standardized to 95% curcuminoids)
S37	local health store	Zagreb, Croatia	processed botanical form	raw material	organically grown turmeric
S38	local health store	Diepholz, Germany	processed botanical form	raw material	100% organically grown turmeric
S39	local health store	Zagreb, Croatia	processed botanical form	raw material	ground turmeric rhizome
S40	local health store	Zagreb, Croatia	processed botanical form	raw material	ground turmeric rhizome
S41	local health store	AL Hoorn, the Netherlands	processed botanical form	hard capsules	turmeric root powder (200 mg), ginger root powder (160 mg), black pepper fruit extract (25 mg)
S42	online supplier	Chiang Mai, Thailand	processed botanical form	hard capsules	*Curcuma longa* (450 mg), *Piper nigrum* (50 mg)
S43	online supplier	Bangkok, Thailand	processed botanical form	hard capsules	turmeric powder
S44	local health store	AL Hoorn, the Netherlands	processed botanical form	herbal tea	organically grown turmeric (49%), verbena (31%), lemon bark (15%), black pepper (5%)
S45	local health store	Wolkersdorf, Austria	processed botanical form	raw material	ground turmeric
S46	local health store	Virovitica, Croatia	processed botanical form	raw material	100% ground turmeric
S47	local health store	Pfaffenhofen, Germany	processed botanical form	tablets	turmeric root, black pepper fruit
S48	local health store	Zagreb, Croatia	processed botanical form	powder bulk	ground turmeric rhizome
S49	online supplier	Patras, Greece	botanical product	raw material	Indian frankincense resin
S50	online supplier	Turkey	botanical product	raw material	Indian frankincense resin
S51	online supplier	not stated	botanical product	raw material	Indian frankincense resin
S52	online supplier	Sonipat, India	processed botanical form	powder bulk	green chiretta herb
S53	online supplier	Rohtak, India	processed botanical form	powder bulk	green chiretta herb
S54	online supplier	Bangkok, Thailand	processed botanical form	hard capsules	green chiretta herb (500 mg, min. 6% andrographolides)

**Table S2:** Results of selectivity testing by slope comparison

Analyte	Slope (direct calibration) *N*=5	Slope (standard addition) *N*=5	Standard error of slope (direct calibration)	Standard error of slope (standard addition)	Correlation coefficient (direct calibration)	Correlation coefficient (standard addition)	*t*-value (*t*_critical_=2.447)	p-value
ANDR	9.80	9.53	0.09	0.13	0.9999	0.9997	1.690	0.141
NANDR	7.42	7.17	0.07	0.09	0.9999	0.9997	2.166	0.073
14-DANDR	7.39	7.27	0.10	0.13	0.9997	0.9995	0.695	0.513
PIP	31.59	31.92	0.44	0.71	0.9997	0.9993	0.391	0.709
BDMC	28.10	27.46	0.52	0.65	0.9999	0.9996	1.242	0.260
DMC	39.05	37.70	0.34	0.53	0.9999	0.9997	2.154	0.075
CUR	37.69	36.63	0.31	0.49	0.9999	0.9937	1.835	0.116
KBA	6.46	6.06	0.06	0.39	0.9999	0.9997	1.003	0.355
AKBA	6.51	6.31	0.06	0.09	0.9999	0.9997	1.804	0.121
ABA	2.71	2.57	0.02	0.16	0.9999	0.9944	0.874	0.416
BBA	2.70	2.76	0.05	0.04	0.9995	0.9997	0.930	0.388

ANDR=andrographolide, NANDR=neoandrographolide, 14-DANDR=14-deoxy-11,12-didehydroandrographolide, PIP=piperine, BDMC=bisdemethoxycurcumin, DMC=demethoxycurcumin, CUR=curcumin, KBA=11-keto-β-boswellic acid, AKBA=3-*O*-acetyl-11-keto-β-boswellic acid, ABA=α-boswellic acid, BBA=β-boswellic acid

**Table S3:** Plackett-Burman design of robustness testing

Standard	A sonication time/min	B dummy factor 1	C extraction temperature/°C	D dummy factor 2	E ethanol ratio in extraction solvent/%	F dummy factor 3	G mobile phase flow/ (mL/min)	H column temperature/ °C	I dummy factor 4	J detection wavelength change/nm^a^	K gradient change/%^a^
12	27	−1	55	−1	77.5	−1	0.95	38.0	−1	−2	−1
1	33	−1	65	−1	77.5	1	1.05	42.0	−1	2	−1
8	33	1	65	1	77.5	−1	0.95	38.0	1	2	−1
16	30	0	60	0	81.5	0	1.00	40.0	0	0	0
3	33	1	55	−1	85.5	−1	1.05	38.0	−1	2	1
7	33	1	55	−1	77.5	1	0.95	42.0	1	−2	1
9	33	−1	65	1	85.5	1	0.95	38.0	−1	−2	1
6	27	−1	55	1	77.5	1	1.05	38.0	1	2	1
5	27	1	55	1	85.5	1	0.95	42.0	−1	2	−1
2	27	−1	65	−1	85.5	−1	0.95	42.0	1	2	1
13	30	0	60	0	81.5	0	1.00	40.0	0	0	0
10	27	1	65	−1	85.5	1	1.05	38.0	1	−2	−1
11	33	−1	55	1	85.5	−1	1.05	42.0	1	−2	−1
4	27	1	65	1	77.5	−1	1.05	42.0	−1	−2	1
14	30	0	60	0	81.5	0	1.00	40.0	0	0	0
15	30	0	60	0	81.5	0	1.00	40.0	0	0	0

^a^Change (nm or percentage of acetonitrile in mobile phase) in relation to standard method conditions, *i.e*. designated wavelengths and gradient program

**Table S4:** Responses of yield of analytes in mixtures and resolution of chromatographic peaks (*R*_s_) in robustness testing

Standard	*Y*(analyte)/(mg/g)										
ANDR	NANDR	14-DANDR	PIP	BDMC	DMC	CUR	KBA	AKBA	ABA	BBA
12	7.73	1.13	3.18	6.88	1.92	1.47	3.59	0.44	3.94	8.29	30.07
1	6.86	0.75	2.78	6.72	1.75	1.39	3.43	0.36	3.26	4.93	19.07
8	7.11	0.87	2.88	6.77	1.73	1.45	3.62	0.42	3.68	6.11	21.09
16	7.33	1.06	2.93	6.98	1.87	1.44	3.55	0.40	3.53	6.12	23.58
3	6.77	0.72	2.65	6.27	1.63	1.33	3.40	0.36	3.29	5.31	19.17
7	7.81	1.06	3.16	6.88	1.91	1.44	3.52	0.44	3.94	7.66	30.46
9	7.69	0.98	3.12	6.54	1.77	1.43	3.58	0.43	3.96	8.45	30.52
6	6.53	0.68	2.60	5.89	1.73	1.31	3.26	0.35	3.14	5.05	18.40
5	7.14	0.83	2.94	6.97	1.83	1.43	3.53	0.39	3.59	5.53	21.23
2	7.40	0.86	3.05	6.93	1.75	1.40	3.53	0.39	3.59	5.49	21.07
13	7.28	1.00	2.93	6.85	1.80	1.41	3.48	0.38	3.53	6.05	23.50
10	6.80	0.89	2.82	6.27	1.69	1.33	3.25	0.40	3.55	7.32	27.56
11	6.75	0.82	2.75	6.05	1.72	1.31	3.15	0.38	3.48	6.89	27.46
4	7.08	0.73	2.93	6.23	1.77	1.32	3.22	0.40	3.54	6.94	27.74
14	7.23	0.97	2.92	6.92	1.85	1.41	3.48	0.39	3.59	6.20	24.09
15	7.14	0.93	2.84	6.80	1.71	1.37	3.38	0.40	3.56	6.10	23.53
Standard	*R*_s_										
	ANDR	NANDR	14-DANDR	PIP	BDMC	DMC	CUR	KBA	AKBA	ABA	BBA
12	2.31	4.52	5.08	2.38	3.69	1.95	1.95	1.27	1.74	2.54	0.97
1	2.51	3.83	4.56	2.34	3.42	2.11	2.11	2.52	1.91	2.35	0.79
8	2.42	4.52	5.11	2.37	4.74	1.94	1.94	1.31	1.72	2.52	0.97
16	1.54	3.81	4.65	2.27	4.06	2.03	2.03	2.09	1.82	2.47	0.89
3	1.51	4.09	4.65	2.03	4.36	1.87	1.90	1.43	1.71	2.50	0.94
7	1.69	3.89	4.66	2.16	2.83	2.06	2.10	2.38	1.94	2.41	0.82
9	1.53	4.10	4.74	2.08	4.46	1.89	1.91	1.23	1.72	2.52	0.96
6	1.53	4.04	4.73	2.12	2.18	1.90	1.92	1.45	1.70	2.48	0.94
5	2.57	3.68	4.70	2.30	3.81	2.12	2.12	2.42	1.93	2.42	0.82
2	1.61	3.98	4.45	2.08	3.78	2.04	2.07	2.35	1.92	2.41	0.82
13	1.27	3.79	4.67	2.26	4.56	2.03	2.03	2.10	1.82	2.47	0.88
10	2.08	4.15	4.82	2.32	4.65	1.93	1.92	1.45	1.71	2.48	0.94
11	1.67	3.68	4.50	2.32	3.51	2.12	2.12	2.56	1.91	2.33	0.78
4	1.62	3.82	4.49	2.10	3.10	2.05	2.08	2.52	1.91	2.32	0.77
14	1.81	2.55	4.64	2.21	3.10	2.02	2.02	2.11	1.82	2.46	0.89
15	1.30	3.84	4.63	2.20	4.50	2.02	2.02	2.14	1.82	2.47	0.88

ANDR=andrographolide, NANDR=neoandrographolide, 14-DANDR=14-deoxy-11,12-didehydroandrographolide, PIP=piperine, BDMC=bisdemethoxycurcumin, DMC=demethoxycurcumin, CUR=curcumin, KBA=11-keto-β-boswellic acid, AKBA=3-*O*-acetyl-11-keto-β-boswellic acid, ABA=α-boswellic acid, BBA=β-boswellic acid

**Table S5:** Factor effects and estimated *E*_critical_ values

Response	A sonication time/min	B dummy factor 1	C extraction temperature/°C	D dummy factor 2	E ethanol ratio in extraction solvent/%	F dummy factor 3	G mobile phase flow/ (mL/min)	H column temperature/°C	I dummy factor 4	J detection wavelength/nm	K gradient change/%	*E*_critical_ (α=0.05)	*E*_critical_ (α=0.01)
*Y*(analyte)/(mg/g)													
ANDR	0.051	−0.042	−0.004	−0.180	−0.010	−0.003	−0.680	0.068	−0.150	−0.340	0.150	0.254	0.446
NANDR	−0.012	0.005	−0.006	−0.060	−0.043	−0.013	−0.170	−0.011	−0.017	−0.170	−0.019	0.068	0.120
14-DANDR	−0.030	−0.014	0.007	−0.070	−0.033	−0.003	−0.300	0.059	−0.058	−0.180	0.026	0.099	0.174
PIP	0.010	0.064	0.062	−0.250	−0.055	0.019	−0.590	0.190	−0.130	0.120	−0.150	0.309	0.543
BDMC	−0.032	−0.013	−0.014	−0.017	−0.072	0.027	−0.110	0.042	−0.022	−0.061	−0.012	0.044	0.077
DMC	0.016	−0.002	0.003	−0.019	−0.025	0.007	−0.100	−0.005	−0.023	0.003	−0.025	0.033	0.058
CUR	0.052	0.001	0.013	−0.057	−0.037	0.011	−0.270	−0.054	−0.070	0.079	−0.010	0.097	0.170
KBA	0.005	0.011	0.008	−0.004	−0.009	−0.003	−0.042	−0.006	−0.001	−0.040	−0.004	0.013	0.023
AKBA	0.043	0.038	0.027	−0.029	−0.004	−0.013	−0.410	−0.028	−0.032	−0.310	−0.007	0.063	0.110
ABA	0.120	−0.036	0.078	−0.005	−0.001	−0.019	−0.850	−0.520	−0.160	−2.190	−0.030	0.176	0.309
BBA	0.290	0.110	0.120	−0.160	0.027	0.110	−2.510	0.039	−0.290	−8.970	0.150	0.390	0.686
*R*_s_													
ANDR	−0.065	0.120	0.160	−0.062	−0.190	0.130	−0.200	0.048	−0.180	0.210	−0.680	0.277	0.487
NANDR	−0.013	0	−0.140	−0.100	−0.160	−0.150	−0.180	−0.420	0.037	−0.003	−0.077	0.196	0.345
14-DANDR	−0.008	0.062	−0.025	0.008	−0.013	−0.012	−0.160	−0.300	0.008	−0.015	−0.180	0.068	0.120
PIP	0	−0.007	−0.016	−0.003	−0.006	0.007	−0.023	0	0.023	−0.020	−0.240	0.027	0.047
BDMC	0.350	0.410	−0.020	−0.160	0.770	−0.300	−0.350	−0.600	−0.190	0.008	−0.520	0.602	1.509
DMC	0	−0.007	−0.007	0.010	−0.007	0.007	−0.003	0.170	0	−0.003	−0.060	0.015	0.026
CUR	0.003	−0.003	−0.009	0.007	−0.009	0.003	−0.007	0.180	0	−0.003	−0.030	0.009	0.016
KBA	−0.005	0.022	−0.004	0.015	−0.002	0.002	0.160	1.100	0.018	0.012	−0.028	0.034	0.060
AKBA	0	0.003	−0.003	−0.007	−0.003	0	−0.200	0.200	−0.003	−0.007	−0.003	0.009	0.015
ABA	−0.003	0.003	−0.007	−0.017	0.007	0.007	−0.060	−0.130	−0.003	0.013	0	0.020	0.035
BBA	0	0	0.001	−0.007	0	0.003	−0.033	−0.150	0.003	0.007	−0.003	0.009	0.015

*R*_s_=resolution of chromatographic peaks, *E*_critical_=critical effect for a response, ANDR=andrographolide, NANDR=neoandrographolide, 14-DANDR=14-deoxy-11,12-didehydroandrographolide, PIP=piperine, BDMC=bisdemethoxycurcumin, DMC=demethoxycurcumin, CUR=curcumin, KBA=11-keto-β-boswellic acid, AKBA=3-*O*-acetyl-11-keto-β-boswellic acid, ABA=α-boswellic acid, BBA=β-boswellic acid

**Table S6:** Factor effects for each response

Response	Significant factors (α=0.05) from comparison with critical effects from negligible effects	Significant factors (α=0.01) from comparison with critical effects from negligible effects	Significant factors from Pareto chart (above the Bonferroni limit, α=0.05)	Important factors from half-normal probability plot
*Y*(analyte)/(mg/g)				
ANDR	G+J	G	G+J	G+J
NANDR	G+J	G+J	G+J	D+E+G+J
14-DANDR_yield_	G+J	G+J	G+J	G+J
PIP	G	G	D+G	G
BDMC	E+G+J	G	/	G
DMC	G	G	/	G
CUR	G	G	G	G
KBA	G+J	G+J	B+C+E+G+H+J	G+J
AKBA	G+J	G+J	G+J	G+J
ABA	G+H+J	G+H+J	A+G+H+I+J	G+H+J
BBA	G+J	G+J	G+J	G+J
*R*_s_				
ANDR	K	K	K	K
NANDR	H	H	/	/
14-DANDR	G+H+K	G+H+K	B+E+G+H+K	B+E+G+H+K
PIP	K	K	E+K	E+K
BDMC	E	/	/	/
DMC	H+K	H+K	H+K	H+K
CUR	H+K	H+K	H+K	H+K
KBA	G+H	G+H	G+H	G+H
AKBA	G+H	G+H	G+H	G+H
ABA	G+H	G+H	D+G+H	G+H
BBA	G+H	G+H	G+H	G+H

*R*_s_=resolution of chromatographic peaks, ANDR=andrographolide, NANDR=neoandrographolide, 14-DANDR=14-deoxy-11,12-didehydroandrographolide, PIP=piperine, BDMC=bisdemethoxycurcumin, DMC=demethoxycurcumin, CUR=curcumin, KBA=11-keto-β-boswellic acid, AKBA=3-*O*-acetyl-11-keto-β-boswellic acid, ABA=α-boswellic acid, BBA=β-boswellic acid

**Table S7:** Non-significant intervals for significant factors of selected responses

Response	Factor	Non-significant interval
*Y*(analyte)/(mg/g)		
ANDR	G	0.981–1.019
	J	−1.5–1.5
NANDR	G	0.980–1.020
	J	−0.8–0.8
14-DANDR	G	0.983–1.017
	J	−1.1–1.1
PIP	G	0.974–1.026
BDMC	G	0.980–1.020
DMC	G	0.983–1.017
CUR	G	0.982–1.018
KBA	G	0.985–1.015
	J	−0.7–0.7
AKBA	G	0.992–1.008
	J	−0.4–0.4
ABA	G	0.990−1.010
	H	39.32–40.68
	J	−0.2–0.2
BBA	G	0.992–1.008
	J	−0.09–0.09
*R*_s_		
ANDR	K	−0.41–0.41
NANDR	H	39.07–40.93
14-DANDR	G	0.980–1.020
	H	39.55–40.45
	K	−0.38–0.38
PIP	K	−0.11–0.11
DMC	H	39.82–40.18
	K	−0.25–0.25
CUR	H	39.90–40.10
	K	−0.30–0.30
KBA	G	0.990–1.010
	H	39.94–40.06
AKBA	G	0.980–1.020
	H	39.91–40.09
ABA	G	0.983–1.017
	H	39.69–40.31
BBA	G	0.986–1.014
	H	39.88–40.12

*R*_s_=resolution of chromatographic peaks, ANDR=andrographolide, NANDR=neoandrographolide, 14-DANDR=14-deoxy-11,12-didehydroandrographolide, PIP=piperine, BDMC=bisdemethoxycurcumin, DMC=demethoxycurcumin, CUR=curcumin, KBA=11-keto-β-boswellic acid, AKBA=3-*O*-acetyl-11-keto-β-boswellic acid, ABA=α-boswellic acid, BBA=β-boswellic acid
